# Intelligent Traffic Control Strategies for VLC-Connected Vehicles and Pedestrian Flow Management

**DOI:** 10.3390/s25226843

**Published:** 2025-11-08

**Authors:** Gonçalo Galvão, Manuela Vieira, Manuel Augusto Vieira, Mário Véstias, Paula Louro

**Affiliations:** 1 Electronics Telecommunication and Computer Department, Instituto Superior de Engenharia de Lisboa, Instituto Politécnico de Lisboa, 1949-014 Lisboa, Portugal; goncalo.galvao@isel.pt (G.G.); manuela.vieira@isel.pt (M.A.V.); mario.vestias@isel.pt (M.V.); paula.louro@isel.pt (P.L.); 2 Department of Electrical and Computer Engineering, School of Science and Technology, Quinta da Torre, Monte da Caparica, 2829-516 Caparica, Portugal; 3 UNINOVA-CTS and LASI, Quinta da Torre, Monte da Caparica, 2829-516 Caparica, Portugal; 4 INESC-INOV, Instituto Superior Técnico, Universidade de Lisboa, 1000-029 Lisboa, Portugal

**Keywords:** deep reinforcement learning (DRL), visible light communication (VLC), multi-agent systems, urban traffic management, autonomous vehicles, traffic management and efficiency

## Abstract

**Highlights:**

Demonstrates adaptive traffic control using Deep Q-Learning in a 5-intersection network. Introduces the SAPA module for dynamic, predictive signal phase adjustments. Integrates VLC-enabled connected vehicles and pedestrian flows for realistic scenarios. SAPA reduces congestion and queue lengths, enhancing overall traffic efficiency. Supports safer, more responsive urban mobility and smarter traffic management.

**What are the main findings?**

- Demonstrates the potential of adaptive deep reinforcement learning strategies to improve urban traffic flow in complex multi-intersection scenarios.
- Highlights how integrating SAPA can further enhance efficiency, reducing congestion and delays for both vehicles and pedestrians.

**What are the implications of the main findings?**

- Adaptive deep reinforcement learning with SAPA can improve urban traffic flow, reducing congestion and delays.
- Cities can achieve more efficient, adaptive traffic management, enhancing mobility and pedestrian safety.

**Abstract:**

Urban traffic congestion leads to daily delays, driven by outdated, rigid control systems. As vehicle numbers grow, fixed-phase signals struggle to adapt to real-time conditions. This work presents a decentralized Multi-Agent Reinforcement Learning (MARL) system to manage a traffic cell composed of five intersections, introducing the novel Strategic Anti-Blocking Phase Adjustment (SAPA) module, developed to enable dynamic phase time adjustments. The goal is to optimize arterial traffic flow by adapting strategies to different traffic generation patterns, simulating priority movements along circular or radial arterials, such as inbound or outbound city flows. The system aims to manage diverse scenarios within a cell, with the long-term goal of scaling to city-wide networks. A Visible Light Communication (VLC) infrastructure is integrated to support real-time data exchange between vehicles and infrastructure, capturing vehicle position, speed, and pedestrian presence at intersections. The system is evaluated through multiple performance metrics, showing promising results: reduced vehicle queues and waiting times, increased average speeds, and improved pedestrian safety and overall flow management. These outcomes demonstrate the system’s potential to deliver adaptive, intelligent traffic control for complex urban environments.

## 1. Introduction

Every day, people traveling within cities face considerable delays in reaching their destinations as a direct consequence of traffic congestion. The continuous and accelerated growth of urban populations has inevitably resulted in a substantial increase in the number of vehicles circulating within city environments. This growth exerts additional pressure on existing road infrastructures, leading to longer queues, increased travel times, and a general decline in transportation efficiency. The resulting congestion not only affects individual mobility but also has broader socioeconomic and environmental implications, such as elevated fuel consumption, increased greenhouse gas emissions, and reduced productivity.

Despite these challenges, the evolution of traffic management systems has not kept pace with the rapid changes in mobility demand. The expansion of vehicle fleets and the growing complexity of urban transport networks have not been matched by proportional investment in, or adoption of, intelligent and adaptive traffic control technologies. In many metropolitan areas, including cities such as Lisbon, the traffic management infrastructure remains largely outdated and rigid, relying predominantly on pre-timed, cyclic phase control strategies that operate under fixed schedules regardless of real-time fluctuations in traffic flow.

This lack of adaptivity severely limits the system’s ability to respond effectively to dynamic traffic conditions, such as sudden congestion peaks, accidents, or variations in demand across different times of day. Furthermore, traffic monitoring and data acquisition continue to depend heavily on traditional, fixed-location sensors—such as inductive loops, magnetic detectors, and fixed cameras—whose spatial coverage is restricted and whose technological capabilities often fall short of capturing the granularity and timeliness required for data-driven control approaches. Consequently, the decision-making processes governing traffic light operations remain largely reactive or pre-programmed, rather than predictive and adaptive, highlighting the urgent need for modern, intelligent control frameworks capable of integrating real-time data and optimizing traffic flow dynamically.

Urban intersections represent one of the most critical elements of traffic networks, as they are the primary sources of queuing and congestion propagation. When poorly managed or insufficiently optimized, intersections can significantly degrade overall traffic flow, increase travel times, and compromise both vehicular and pedestrian safety. Considering these challenges, there is a clear need for the development of adaptive traffic control systems capable of dynamically managing the active signal phases at intersections based on real-time traffic data.

The motivation for this work arises from the limitations of existing urban traffic management systems and the growing demand for sustainable and intelligent mobility solutions. Conventional traffic control frameworks are unable to exploit the vast amount of data generated by emerging connected and automated vehicle technologies. At the same time, recent advances in artificial intelligence and wireless communication open new opportunities for the development of adaptive, data-driven control strategies capable of responding to rapidly changing traffic conditions [1]. In particular, the integration of Visible Light Communication (VLC) with Deep Reinforcement Learning (DRL) offers a promising path toward more efficient, reliable, and scalable traffic control architectures [2,3]. VLC-based systems can leverage the existing urban lighting infrastructure for both illumination and high-speed data exchange between vehicles and traffic lights, enabling precise and low-latency communication. Combined with DRL algorithms capable of learning optimal control policies from real-time observations, this approach has the potential to significantly improve intersection performance, reduce congestion, and contribute to the broader vision of intelligent and sustainable urban mobility.

To address this need, we propose an intelligent traffic control system leveraging the DRL framework. This approach enables the system to learn optimal traffic signal strategies through real-time data collection and dynamic phase selection, adapting signal timing to prevailing traffic conditions [4]. By continuously learning from traffic flow patterns, the DRL-based system can optimize vehicular movement along both radial and circular corridors, effectively prioritizing inbound and outbound traffic according to the city’s temporal demand patterns.

For real-time traffic information acquisition, the proposed system integrates emerging vehicular technologies, particularly connected vehicles (CVs), in combination with VLC. Connected Vehicles present a significant opportunity for advanced traffic management, as they can share real-time traffic and safety data with both surrounding vehicles and the roadside infrastructure, thereby enhancing safety, efficiency, and comfort [5]. VLC, an innovative complementary technology, exploits the intensity modulation of Light Emitting Diodes (LEDs)—widely used in streetlights, traffic signals, and vehicle headlights—to transmit data within the urban environment. This dual functionality of LEDs for both illumination and data communication establishes VLC as a key enabler for intelligent intersections, where it can facilitate efficient signal optimization and vehicle trajectory coordination.

This research investigates the integration of VLC and AI, with a focus on DRL-based strategies, for adaptive traffic control. Specifically, we explore the design and training of neural networks capable of determining dynamic phase timings using the SAPA module developed in this study. The system applies DRL principles within a connected vehicular ecosystem to improve intersection performance, optimize arterial traffic flow, and balance the operational needs of both vehicles and pedestrians in real-world urban scenarios. By leveraging VLC and CVs, this work aims to demonstrate the potential of emerging communication and learning technologies to address modern urban traffic management challenges effectively.

The remainder of this manuscript is organized as follows: Section 2 presents a comprehensive literature review and background on the application of VLC and DRL in traffic management, as well as the role of connected vehicles. Section 3 details the methodology of the proposed V-VLC system integrated with a Multi-Agent Reinforcement Learning framework. Section 4 presents the performance analysis of the trained networks under various traffic strategies. Finally, Section 6 discusses the implications, limitations, and directions for future research.

## 2. Background and Literature Review

### 2.1. Overview of Urban Mobility Challenges

Urban traffic management increasingly struggles with congestion, delays, and safety issues as growing populations and vehicle volumes place heavy demands on limited infrastructures. Intersections often become bottlenecks, particularly during peak hours, while accidents and outdated signal systems further intensify delays. The interaction among pedestrians, cyclists, public transport, and private vehicles in shared spaces adds further complexity to traffic flow in dense urban areas.

Congestion is largely worsened by static signal control that cannot adjust to real-time conditions, leading to unnecessary stops and long queues. The absence of coordination between neighboring intersections also disrupts overall flow and efficiency [6,7]. Traditional strategies such as road widening or fixed-time signals are reactive and unsustainable, failing to meet the dynamic needs of modern mobility. Emerging solutions—like adaptive traffic control, connected vehicles, and intelligent communication networks—show greater potential [7,8]. However, most remain vehicle-centered and overlook pedestrian activity. Integrating pedestrian behavior into reinforcement learning–based control systems is essential to balance efficiency, safety, and inclusiveness in urban environments.

In multi-intersection contexts, single-agent control approaches face scalability limitations, prompting research into collaborative strategies. Recent studies have introduced mechanisms that consider factors such as queue length at neighboring intersections and the interdependencies among them, seeking to achieve both efficiency and scalability in large-scale networks [8]. Building on these developments, our proposed adaptive traffic control framework leverages real-time and predictive traffic flow data within Vehicle-to-Everything (V2X) environments to enhance both operational efficiency and safety [9,10]. In contrast to conventional systems that depend on fixed detectors and provide only limited data on traffic occupancy and flow, V2X-based adaptive systems enable a more comprehensive and dynamic perception of road conditions. They collect detailed information such as vehicle speed, position, queue length, and stop duration, allowing for more informed and flexible control decisions. While Vehicle-to-Vehicle (V2V) communication supports safety functions like collision avoidance and pre-crash detection, Infrastructure-to-Vehicle/Pedestrian (I2V/P) interaction extends this framework by delivering relevant, real-time information to both drivers and pedestrians. Collectively, these communication modes foster a more coordinated and intelligent traffic management environment that promotes efficiency, adaptability, and safety.

### 2.2. Vehicular Visible Light Communication Integration and Challenges

Visible Light Communication (VLC) is emerging as a promising component in the evolution of Intelligent Transport Systems (ITS), with potential applications in areas such as signalized intersections, collision warning and avoidance, vehicle localization and detection, and vehicle platooning. These applications are supported by different communication modes, including V2V, infrastructure-to-vehicle (I2V), and vehicle-to-everything (V2X) links [11].

Although radio frequency (RF) communication is a cornerstone of ITS, it presents several limitations in dense urban environments. Problems such as electromagnetic interference, restricted spectrum availability, and vulnerability to security attacks often compromise its efficiency. The presence of electrical devices and multiple wireless systems intensifies electromagnetic noise, while the growing demand for RF spectrum contributes to congestion. Moreover, the open nature of RF signals makes them more susceptible to interception and hacking attempts. VLC, on the other hand, leverages LED-based light modulation for data transfer and offers clear advantages: it is immune to electromagnetic interference, operates in an unlicensed spectrum, reduces spectrum congestion, and provides enhanced security. Furthermore, by relying on existing lighting infrastructure, VLC presents a cost-effective solution [12].

Despite these benefits, VLC also faces challenges in outdoor scenarios due to environmental factors [13]. Conditions such as intense sunlight, fog, rain, and snow can attenuate light signals, while direct sunlight may saturate optical receivers, leading to performance degradation [14]. For this reason, VLC should not be seen as a replacement for RF, but rather as a complementary technology. Combining the two can yield more robust and reliable communication systems: VLC offers high-capacity data exchange through line-of-sight (LoS) links, while RF ensures connectivity in non-line-of-sight (NLoS) conditions or when adverse weather reduces VLC effectiveness.

Recent studies have examined the impact of outdoor environmental conditions on VLC performance, particularly in terms of bit error rate (BER), as well as the influence of inter-vehicle distance. In ref. [15], a VLC system for tunnels is proposed, focusing on I2V communication and considering not only the typical LoS channel from infrastructure transmitters but also NLoS components, including reflections from tunnel walls. Similarly, ref. [16] provides a detailed investigation of V2V VLC, analyzing diverse environmental conditions, varying lateral distances between vehicles, and the influence of ambient light during both daytime and nighttime scenarios. The results are promising, demonstrating the robustness and capacity of VLC even under challenging conditions. Nonetheless, performance degradation is observed under dense fog, high ambient light intensity, or large inter-vehicle distances, highlighting the limitations of the technology in extreme outdoor scenarios.

While VLC provides substantial advantages for short-range, high-capacity communication in dense urban environments, it also presents intrinsic challenges. Because data transmission relies on light modulation, simultaneous emissions from multiple transmitters (e.g., vehicles and infrastructure units) can cause interference and data collisions, especially in heavy-traffic scenarios. Such interference can degrade signal quality and reliability, potentially compromising real-time vehicular communications. To mitigate these effects, several techniques have been proposed, including Optical Code Division Multiple Access (OCDMA) to reduce multi-user interference [17], advanced modulation and adaptive power control schemes to enhance link stability, and hybrid VLC/RF architectures that dynamically switch between optical and radio channels depending on network and environmental conditions [18,19,20].

In the context of Connected Autonomous Vehicles (CAVs), integrating VLC into both vehicles and traffic light systems can significantly enhance traffic management. Through V2I and I2V communication, traffic lights equipped with VLC could dynamically adapt their active phases and timings to real-time traffic flow, helping reduce congestion, waiting periods, and overall travel time. On the vehicle side, V2V communication would enable the exchange of critical information such as road conditions, accident alerts, optimal routes, and vehicle status. It could also share lane positions, speed, trajectory, and data obtained from VLC-equipped roadside infrastructure like smart lighting. Notably, in high-density traffic scenarios, VLC’s effectiveness increases, as reduced inter-vehicle distances facilitate chain-like communications (V2V2V2V2I2V). This interconnected environment fosters seamless coordination between vehicles and infrastructure, moving toward a fully connected traffic ecosystem.

Building upon these developments, this paper introduces a novel framework that integrates VLC-based localization services with learning-driven traffic signal control, aiming to optimize both vehicular and pedestrian mobility across multi-intersection networks. The core objective is to minimize waiting times while enhancing traffic safety.

To achieve this, reinforcement learning (RL) techniques are employed to manage the complex interactions at intersections through V2V, vehicle/pedestrian-to-infrastructure (V/P2I), and infrastructure-to-vehicle/pedestrian (I2V/P) communication. Within this paradigm, agents learn decision-making strategies by receiving rewards for effective actions and penalties for less favorable ones [21,22]. The proposed VLC-enabled intelligent control model is assessed using the Simulation of Urban MObility (SUMO) platform, which provides an agent-based simulation environment [23,24]. In this setup, traffic lights operate as learning agents that continuously observe traffic conditions, adapt their strategies through RL, and progressively improve their performance to ensure smoother and safer mobility.

### 2.3. Deep Reinforcement Learning in Traffic Control Systems

Traffic management plays a crucial role in maintaining the efficiency of urban mobility systems. Without effective planning and control, road networks are prone to severe congestion, resulting in longer travel times and significant delays. With the advent of artificial intelligence, Deep Reinforcement Learning (DRL) has emerged as a powerful tool for vehicular communication and traffic optimization. In particular, DRL leverages deep neural networks (DNNs) to recognize and interpret complex traffic dynamics, enabling more adaptive and intelligent traffic signal control strategies [25,26].

In DRL-based systems, state observation can be performed by one or multiple agents, depending on the complexity of the traffic environment. Research in this area spans from relatively simple scenarios involving a single intersection—typically managed by a single reinforcement learning (RL) agent—to more complex multi-intersection networks, which fall under the domain of Multi-Agent Reinforcement Learning (MARL). Within MARL, several key properties shape system design: whether control is centralized or decentralized, whether the environment is fully or partially observable, and whether interactions are cooperative or competitive. In centralized approaches, a single controller determines the actions for all agents at each time step, while in decentralized settings, each agent independently selects its own action. Furthermore, agents may cooperate to achieve a shared objective or act competitively to maximize individual rewards. Their perception of the environment may be restricted to local information or expanded through communication with neighboring agents [27].

Formally, RL environments are often modeled as Markov Decision Processes (MDPs), represented as a five-tuple <S, A, P, R, γ> [28]. Here, S denotes the finite set of states, A the set of actions, P the transition probability matrix, R the reward function, and γ ∈ [0, 1] the discount factor balancing the importance of immediate and future rewards. At each time step t, an agent observes the current state st, executes an action at, transitions to a new state s_t+1_, and receives a reward rt. Positive rewards reinforce the likelihood of repeating certain behaviors, while negative rewards discourage them. The ultimate goal is to maximize the cumulative discounted reward over time [29].

For traffic optimization problems, modeling choices vary with the type of environment, its representation, and the design of reward functions, neural network architectures, and learning algorithms. In ref. [30], for example, the environment consists of a single intersection, where the system state is defined by vehicle queues and waiting times on each lane. The reward function is based on the average waiting time of all vehicles, while the action space comprises four traffic signal phases: North–South straight, North–South left turn, West–East straight, and West–East left turn. In ref. [31] a wireless-powered mobile edge computing network with multiple hybrid access points (HAPs) was analyzed. Wireless devices harvest energy from RF signals transmitted and then process their computation tasks locally or offload them to selected HAPs. They propose a framework, which consists of a high-level agent at HAPs and multiple low-level agents at the mobile devices, to solve this problem in a distributed manner. In ref. [32,33] a PPO Actor–Critic algorithm is used for both learning and decision-making. This approach also enables communication between agents during training, allowing them to exchange traffic observations. The results demonstrate improved learning efficiency, enhanced stability and generalization, and faster convergence, with performance evaluated through metrics such as average waiting times and vehicle arrival rates.

In ref. [34], a more complex four-intersection network is studied, requiring a MARL approach. Roads are divided into small cells, each indicating the presence of a vehicle and its normalized speed. Similar to the previous work, four traffic signal phases are considered, but here the reward function is based on queue lengths. Each intersection is controlled by an individual agent using its own neural network, while Cooperative Q-Learning facilitates information sharing between neighboring intersections. The results show strong performance across multiple metrics, including average speeds, queue lengths, and waiting times.

Nonetheless, a significant limitation of current state-of-the-art approaches lies in their exclusive focus on vehicular flows, with little or no consideration given to pedestrian dynamics. There is a clear need for models capable of integrating both vehicle and pedestrian interactions in order to enhance not only efficiency but also safety within urban traffic systems. Furthermore, the existing literature on traffic signal control has not yet advanced toward the development of intelligent traffic management frameworks designed to create broader traffic strategies that regulate specific urban zones.

Moreover, unlike many studies in the state of the art, this work considers an urban traffic environment fully adapted for connected vehicles, featuring a single through lane and a dedicated left-turn lane on each approach of the intersection. This design aligns with emerging vehicle technologies, ensuring that vehicles are positioned in the appropriate lane prior to crossing the intersection, thereby reducing lane-changing maneuvers, which are among the primary contributors to congestion.

Furthermore, this study emphasizes a more holistic approach to traffic control through intelligent systems, recognizing that urban traffic management cannot be effectively addressed in isolation at individual intersections but requires cooperative coordination among all agents. In this context, cooperative traffic strategies are developed to handle diverse traffic scenarios that reflect real-world daily conditions. When discussing city-wide traffic control, it is essential to recognize that traffic management is inherently strategy-based—this is a fundamental concept in traffic control systems. Even at a single intersection, different strategies can be applied to prioritize one direction over another or to maintain balance between conflicting flows. As the controlled area expands—in this case, as the number of interconnected intersections increases—the role of such strategies becomes increasingly significant. Adaptive DRL strategies act as intelligent coordination frameworks among distributed agents operating within the same traffic environment. Through continuous learning and policy refinement, these agents collectively improve traffic flow, enhance responsiveness to real-time fluctuations, and contribute to a more sustainable and efficient urban mobility system in complex multi-intersection scenarios.

This paper contributes to addressing this gap by presenting a comprehensive perspective on urban traffic control, with a focus on adaptive strategies based on DRL. The proposed framework leverages DRL-trained models to implement these strategies [35], enabling more flexible and effective management of urban traffic. A thorough analysis is conducted across both radial and circular arterial intersections, as well as major city entry and exit points, providing a deeper understanding of traffic dynamics across different spatial scales and facilitating more coordinated and strategic control of urban mobility.

## 3. Proposed Framework and Problem Statement

### 3.1. Vehicular Connectivity Through Visible Light Communication

Connected Vehicle (CV) technologies are fundamentally transforming urban traffic management by enabling continuous information exchange both between vehicles (V2V) and with roadside infrastructure (V2I). Through this connectivity, vehicles are able to share real-time data—such as position, speed, and road conditions—which supports traffic flow optimization, congestion mitigation, and improved safety. Consequently, CVs are increasingly recognized as a key enabler of next-generation Intelligent Transportation Systems (ITS).

In parallel, Visible Light Communication (VLC) has emerged as a complementary technology with strong potential for traffic management. VLC operates by modulating the intensity of LED-based light sources, such as traffic signals and streetlamps, to transmit data while preserving their primary illumination function. This dual functionality enables seamless integration into existing urban infrastructure, effectively transforming the lighting grid into a large-scale communication backbone.

**Figure 1:**
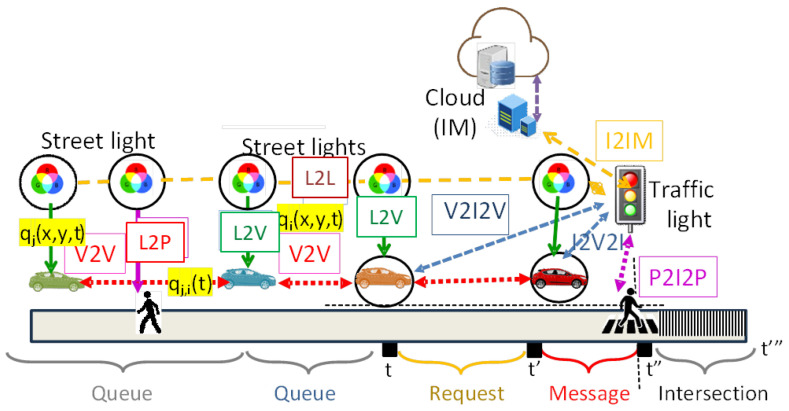
A 2D representation of the V-VLC architecture [14].

The proposed V-VLC architecture, illustrated in Figure 1, builds upon this principle by leveraging both vehicles and infrastructure components within a unified Vehicle-to-Everything (V2X) framework. Communication takes place across multiple dimensions: V2V, vehicle-to-infrastructure (V2I), and infrastructure-to-vehicle (I2V). Furthermore, the model explicitly incorporates pedestrian interaction through Pedestrian-to-Infrastructure (P2I) and Infrastructure-to-Pedestrian (I2P) links. In this context, pedestrians can issue crossing requests via VLC-enabled devices, while the infrastructure responds with safe-phase allocations and trajectory guidance.

These communication mechanisms are directly embedded into a Multi-Agent Reinforcement Learning (MARL) framework, ensuring that both vehicular and pedestrian flows are dynamically accounted for in the signal control process. To regulate intersection traffic, a queue–request–response protocol is employed: approaching vehicles and pedestrians submit crossing requests (V/P2I), and the traffic lights, acting as intelligent agents, acknowledge these requests (I2V/P). Signal phases are then adaptively adjusted to resolve potential conflicts, thereby optimizing throughput while simultaneously enhancing pedestrian safety.

Within the V-VLC architecture, a hybrid mesh–cellular design is adopted. Streetlights act as “mesh” nodes to relay information to vehicles, while traffic lights employ a hybrid mesh/cellular controller that performs edge computing tasks.

Naturally, outdoor deployments face environmental challenges such as fog, rain, snow, and interference from sunlight or artificial lighting. However, advances in photodetectors, filters, emitters, and LED technology significantly reduce these limitations. In extreme cases where VLC cannot function reliably, radio frequency (RF) communication serves as a backup, ensuring system robustness.

**Figure 2:**
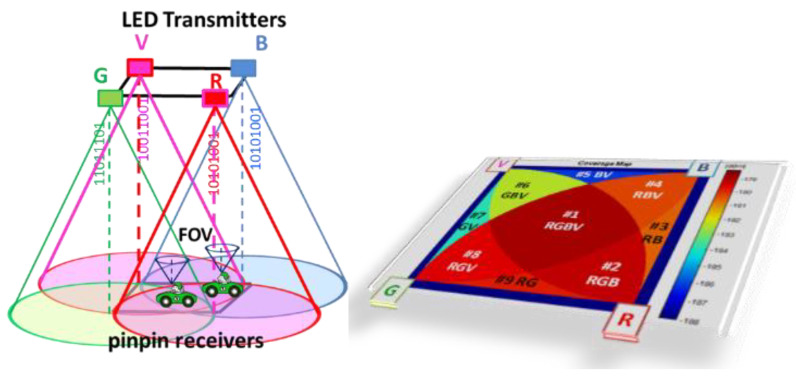
V-VLC emitter and receivers’ relative position and an illustration of the coverage map, with the footprint regions in the unit cell (#1#9) [12].

The VLC system comprises a modulated light transmitter and a PIN–PIN-based photodetector that captures light intensity variations [36]. Their interaction is illustrated in Figure 2, which shows emitter–receiver positioning, the coverage footprint of each unit cell (#1–#9). Modulation is implemented using ON–OFF keying (OOK). Each square unit cell is equipped with tetra-chromatic white LED (WLED) sources at its corners. These WLEDs are composed of four chips—red (626 nm), green (530 nm), blue (470 nm), and violet (390 nm)—which combine to produce white light.

Because each VLC infrastructure unit operates with four independent emitters, the received optical signal may consist of one, two (#3, #5, #7, #9), three (#2, #4, #6, #8), or four (#1) excitations, creating 24 possible optical combinations and 16 distinct photocurrent outputs at the detector. A PIN–PIN demultiplexer is used to separate these signals, leveraging prior calibration knowledge to correctly interpret the encoded OOK amplitudes and reconstruct the transmitted data.

### 3.2. Urban Traffic Scenario

For the assessment and development of adaptive traffic control systems, it is essential to clearly define the environment in which intersections are situated, as well as the specific type of intersection under analysis. Such characterization provides a fundamental understanding of the conditions under which traffic flows through these junctions and underscores how an accurate definition of the intersection type influences overall traffic dynamics and system performance.

**Figure 3:**
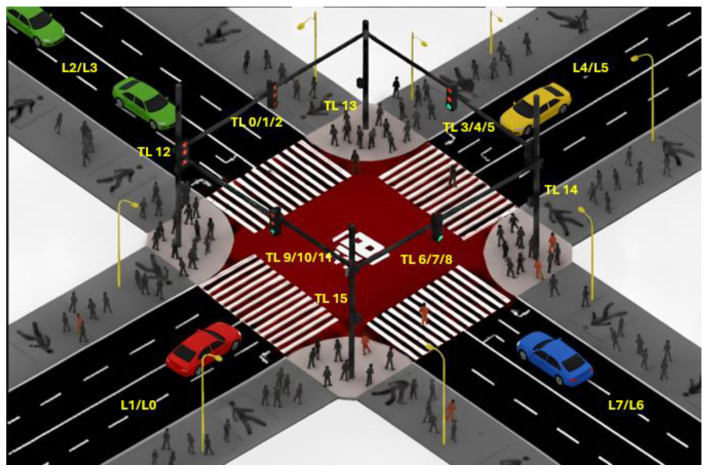
Schematic diagram of a signal controlled standard intersection with coded lanes (L/0-7) and traffic lights (TL/0-15).

The traffic environment considered in this study corresponds to an urban scenario, where both inbound and outbound flows to and from the city are modeled, with peak hours being considered for traffic generation. The layout of the intersections under consideration is illustrated in Figure 3. Unlike many intersection models commonly employed in the state of the art, the present configuration assumes only two lanes per approach, resulting in a total of eight lanes (L/0–7). These lanes are controlled by a total of sixteen traffic lights (TL/0–15). The rightmost lane allows through movements as well as right turns, whereas the left lane is dedicated exclusively to left-turn maneuvers. Pedestrian crossings are also included, which represents a significant departure from several previous studies that disregard pedestrian circulation.

In alternative intersection models that incorporate multiple through lanes, traffic management strategies are strongly affected by the increased storage capacity, as larger queues can be accommodated without immediate spillback, thereby reducing waiting line lengths. By contrast, the intersection analyzed in this work has been specifically designed for Connected Autonomous Vehicles (CAVs). In this context, vehicles are assumed to already be positioned in the appropriate lane before reaching a queue or a signalized stop, thus eliminating the need for unnecessary lane changes and enabling more efficient traffic flow management.

**Figure 4:**
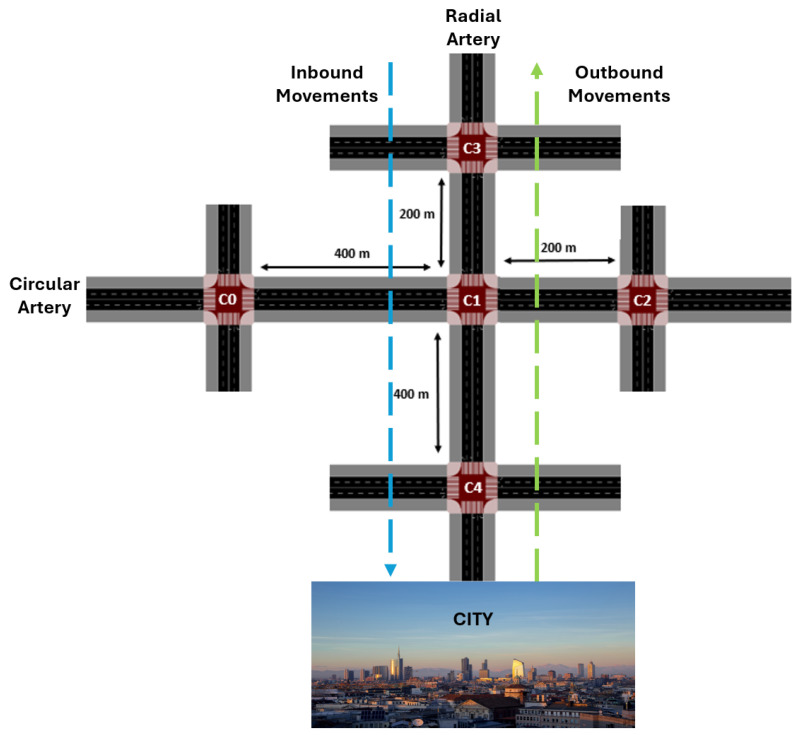
Traffic Scenario consisting of 5 homogeneous intersections. Arrows are ascribed to the inbound and outbound movements directions.

Having examined in detail the type of intersection and the traffic environment under study, we now extend the analysis to the global traffic scenario, which comprises five intersections, as illustrated in Figure 4. The network is modeled as a simplified urban traffic system consisting of two intersecting arterial roads: circular artery (C0–C1–C2) and a radial artery (C3–C1–C4), which converge at the central junction C1.

Intersection C1 functions as the sole connection point between both arteries. Unlike its neighboring junctions, C1 does not generate local traffic demand; rather, it exclusively receives flows from adjacent intersections. Consequently, the inflows into C1’s lanes are entirely determined by the phase-activation decisions of the neighboring controllers (agents C0, C2, C3, and C4). As such, C1 serves as a critical hub within the network, mediating the interaction between the two arterial streams and exerting a significant influence on overall traffic dynamics. Its coordination can promote balanced traffic dispersal or alleviate congestion; however, if misaligned, it may also introduce systemic imbalances. For this reason, we investigate how different priority schemes imposed at C1 affect traffic flow across the entire network. Inbound and outbound movements are also considered, corresponding to flows from C3 to C4 and from C4 to C3, respectively. This setup simulates the most critical traffic patterns in an urban environment, representing the city’s main entry and exit points during morning and evening peak hours.

Furthermore, the roads connecting the intersections do not all have the same length. For instance, the links between C0–C1 and C4–C1 are 400 m long, whereas those between C3–C1 and C2–C1 measure only 200 m. These differences are intended to reflect the variability of real-world traffic scenarios, where road segments rarely present uniform dimensions. Such heterogeneity also entails implications for queue dynamics: longer links (400 m) provide greater vehicle storage capacity, while shorter links (200 m) are more easily saturated, generating additional pressure on the intersections. Consequently, these segments require more cautious and adaptive management within the control strategy.

**Figure 5:**
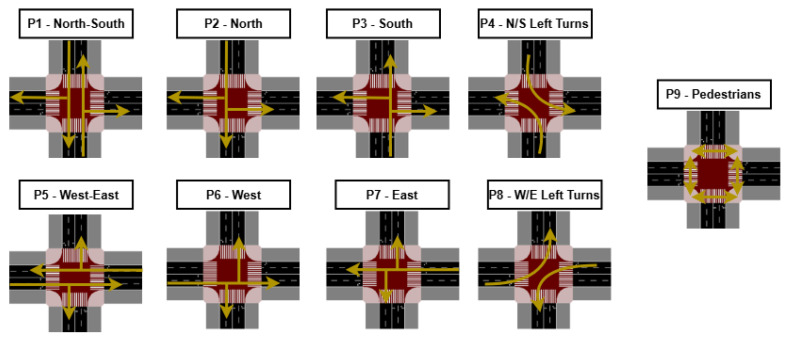
Traffic signal phases considered: eight for vehicles (P1–P8) and one exclusively for pedestrians (P9). The arrows represent the directions of vehicle movements across the eight vehicular phases, while P9 corresponds to the exclusive pedestrian phase at the signalized intersection.

The traffic circulating through these intersections is regulated by signalized control. Each signal system is composed of nine phases in total, as illustrated in Figure 5. These phases are activated intelligently with the objective of minimizing queue lengths and waiting times, thereby optimizing traffic flow at each intersection. Phase activation is determined in real time according to prevailing traffic conditions, which enables greater adaptability and the development of strategies that enhance vehicular throughput. Moreover, this adaptive coordination also accounts for the state of neighboring intersections, preventing them from becoming overloaded due to excessive inflows.

Of the nine total signal phases, eight are allocated to vehicular movements, while a single phase is reserved exclusively for pedestrian crossings. During this pedestrian phase, all vehicular movements are suspended, enabling pedestrians to traverse designated crosswalks safely without conflicts with vehicles. Pedestrian crossings are strictly restricted to their corresponding phase. This configuration not only optimizes traffic flow but also substantially enhances safety for both pedestrians and vehicles.

## 4. DRL Framework for Urban Traffic Control

### 4.1. Multi-Agent Reinforcement Learning System and Network Architecture

**Figure 6:**
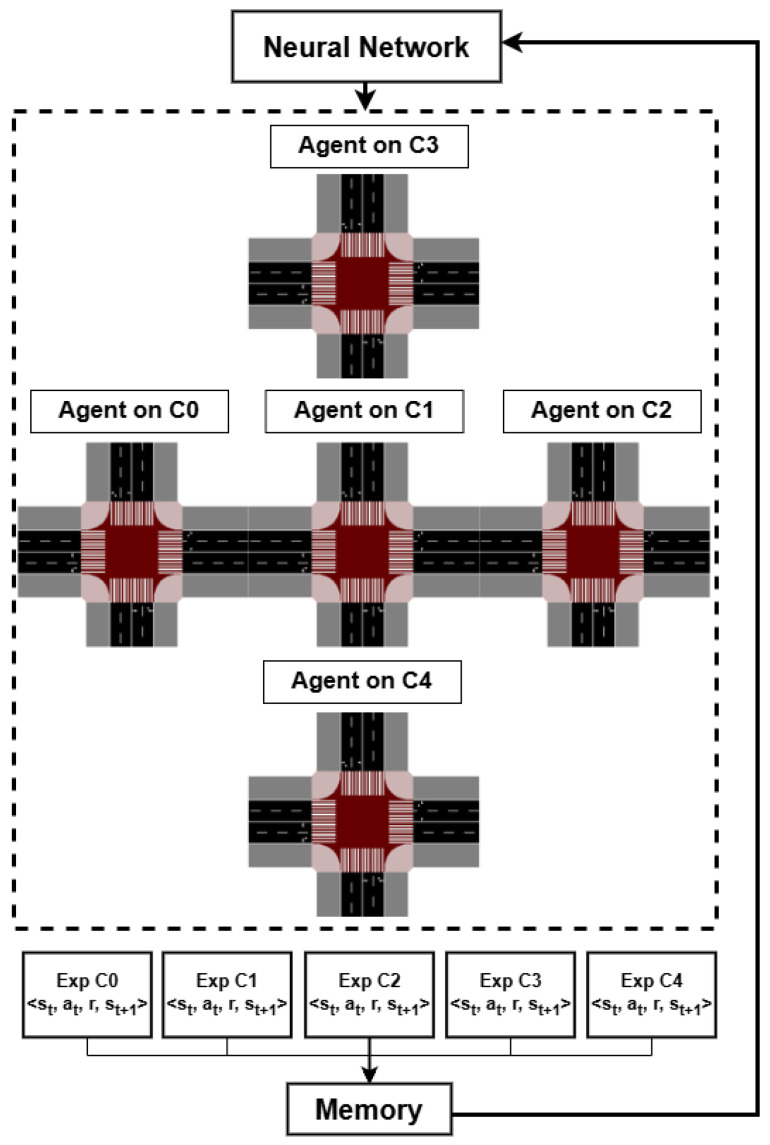
Multi-Agent Reinforcement Learning System with a CTDE architecture.

A proposed multi-agent reinforcement learning system (MARL) is designed to manage the five intersections within the studied urban scenario, as depicted in Figure 6. The system’s architecture leverages a single, shared neural network, with each intersection governed by a distinct agent. Each of these agents is tasked with observing its local environment, specifically the traffic state of all incoming lanes, and subsequently selecting one of nine predefined actions.

The agent’s experience is captured as a tuple: <s_t_, a_t_, r, s_t+1_>. This tuple consists of the current state of the intersection (s_t_), the action taken by the agent (at), the reward received (r), and the resulting future state (s_t+1_). These experiences, collected from each agent, are aggregated into a shared memory buffer. This pooled data is then used to train the singular neural network, a process that enables the agents to learn from the collective experience of the entire system.

This architectural design, known as Centralized Training with Decentralized Execution (CTDE), was adopted to balance efficiency, scalability, and coordination in multi-intersection traffic control. Prior research has shown that this form of indirect cooperation among agents provides substantial benefits for overall network performance. In our system, each of the five agents controls a single intersection while sharing a common neural network and experience replay memory. By centralizing the learning process, the network benefits from aggregated experiences, improving convergence and training efficiency, while enhancing coordination of traffic flow across the arterial network. The homogeneity of the intersections further enriches the training process, as observed experiences tend to be similar across agents. Compared to a fully centralized approach with a unified action space, CTDE avoids combinatorial explosion, reduces computational cost, and allows scalable expansion to additional intersections, while experimental results confirmed faster convergence and robust control under this architecture.

Each intersection is managed by a dedicated MARL agent that perceives its local environment—collecting data on vehicles and pedestrians via VLC-based communication—and cooperates with neighboring agents through shared information. This collected data is then used to train a neural network based on the Deep Q-Learning algorithm. This approach enables the network to learn how to select the optimal signal phase at each intersection by estimating the expected cumulative rewards. Unlike traditional tabular Q-Learning, which is limited to small state-action spaces, the DQN leverages neural networks to handle the complexity and high dimensionality of large-scale urban traffic environments.

The implemented neural network architecture is a Fully Connected Layer Network (FCLN). Its weights, denoted as θ_k_, are adjusted to approximate the Q-value function, Q (s,a;θ_k_), which represents the expected value of an action a in a given state s. The state representation in the proposed architecture comprehensively encodes the traffic environment by combining positional, velocity, and waiting time data.

The input state vector consists of 3 layers. The first one is made up of 80 cells, 10 for each lane, routing vehicles to the junction, indicating their presence. If a vehicle is inside the cell, it is filled with ‘1’; otherwise, ‘0’. The second layer, made up of the same number of cells, indicates the normalized speed of the cars in each cell, if any are present. The third layer, made up of just 4 cells, represents the waiting zones, indicating the number of pedestrians standing still waiting for their phase to become active. This results in a total of 164 neurons in the input layer. The network architecture includes two hidden layers, each with 400 neurons, using the Rectified Linear Unit (ReLU) activation function to introduce non-linearity and facilitate the learning of complex traffic patterns. The output layer is composed of nine neurons, each corresponding to the Q-value of a possible control action, with the Mean Squared Error (MSE) loss function used to optimize the training process.

### 4.2. Deep Q-Learning Algorithm and Composed Reward

The training of the neural network was accomplished using the Deep Q-Learning (DQL) algorithm. This approach is characterized by the use of two distinct neural networks with identical architectures: a primary network responsible for predicting the Q-values (Qpred) and a separate target network, less frequently updated, which is used to compute the target Q-values (Qtarget), as defined in Equation (1).

(1) $$Q_{target}=r_{t}+ϒ\cdot \max\left[Q_{pred}\left(s_{t+1},a\prime ,\theta_{k}\;\right)\right]$$

where Q_pred_ is the Q-value predicted by the main network and Q_target_ is acquired using a network similar to the main one. The target network is not trained via backpropagation; instead, its weights are periodically copied from the main network. This asynchronous update mechanism is crucial for mitigating the correlation between predicted Q-values and the targets, which enhances the stability and convergence of the algorithm. It also incorporates a discount factor *γ* applied to the *maxQ_target_* value, lowering the importance of the future reward compared to the immediate reward.

The reward rt serves as a metric for evaluating the agent’s action in its current state. The reward function employed here is a novel contribution, as it comprises two distinct components: one for vehicles and one for pedestrians. This is a composed reward, as defined in Equations (2) and (3). This design allows the agent to perceive how its chosen action impacts both vehicular and pedestrian traffic. To balance these components, two weights, pped and pveh, are introduced. These weights provide the flexibility to prioritize either vehicles or pedestrians, or to maintain a balanced system. For the purpose of this study, the weights were set to achieve this 50/50 equilibrium.

(2) $$r_{t}=p_{veh}\left(atwt_{veh,t-1}-atwt_{veh,t}\right)+p_{ped}\left(atwt_{ped,\;t-1}-atwt_{ped,t}\right)$$

The reward used considers both vehicle and pedestrian average total accumulated waiting times (atwt). $wt_{veh,t}/wt_{ped,t}$ is the amount of time in seconds a vehicle/a pedestrian has a speed of less than 0.1 m/s at t, since the spawn into the environment. n represents the total number of vehicles/pedestrians in the environment in t.

(3) $$atwt_{veh,t}=\sum_{veh=1}^{n}wt_{\left(veh,t\right)}\;\;{atwt}_{ped,t}=\sum_{ped=1}^{n}{wt}_{\left(ped,t\right)}$$

The reward function implemented in this system, formulated on the basis of weighted vehicle and pedestrian waiting times, is designed to minimize cumulative delays at each time step, thereby providing immediate feedback to the DRL agents for adaptive traffic signal control. Alternative reward structures were also explored, including queue-length reduction, throughput maximization, and hybrid metrics integrating multiple performance objectives. Preliminary experimental results indicate that the selected approach facilitates effective convergence and rapid adaptation to dynamically evolving traffic conditions.

Overall, this approach combines local perception, via V-VLC data, and global cooperation to deliver intelligent, data-driven traffic signal decisions. The result is an efficient solution that operates in real time within multi-agent urban environments, addressing both the complexity and dynamism of modern city traffic.

### 4.3. MARL System with Strategic Anti-Blocking Phase Adjustment

The traffic control framework adopts a decentralized Multi-Agent Reinforcement Learning (MARL) system, where each agent manages its own intersection by observing local conditions, selecting active phases, and storing experiences. Since intersections are homogeneous, these experiences can be shared across agents to train a common neural network. This approach enhances adaptability compared to fixed-cycle systems, as it allows signal phases to respond to real-time traffic conditions.

Another key improvement for this intelligent system is the introduction of dynamic phase duration, supported by data collected through VLC on vehicle queues and lane occupancy. Unlike fixed durations (e.g., 8 or 12 s), phase times are adjusted based on both the number of waiting vehicles and the occupancy of the receiving lanes at neighboring intersections. This micro-level control prevents blockages and optimizes throughput, forming the basis of the SAPA mechanism. This dynamic time allocation mechanism allows the intersection-controlling agent not only to adaptively select the active phase but also to adjust its duration according to the number of vehicles intending to cross during that phase. The duration of each phase can, thus, be modulated to allow higher or lower traffic throughput, depending both on the volume of vehicles waiting to traverse the intersection and on the occupancy rate of the adjacent intersection’s approach. This ensures a more balanced and coordinated traffic flow across neighboring control zones.

Considering the scenario of five intersections under study, let us focus on intersections C0 and C1, which are connected by a 400 m link. This longer road segment provides a higher vehicle storage capacity. For phases that allow vehicle movements towards this link—such as phase 5 (West–East) or phase 6 (West)—the system first evaluates both the occupancy rate ρ, of the 400 m link and the number of vehicles queued at intersection C0. If the occupancy is below 40%, the phase is activated with a green time *T*, adapted to the number of vehicles waiting, according to Equation (4). Otherwise, the phase is assigned only to its minimum duration, which prevents excessive vehicle inflow into the link and allows intersection C1 to reduce its level of congestion.

In this formulation, *T_base_* refers to the base duration of the phase, set to 8 s. *Q* denotes the number of vehicles waiting at the time phases 5/6 are triggered. $\alpha_{rad}$ and $\alpha_{circ}$ is associated with the control strategy applied, which may either prioritize one of the arteries (65% or 35%) or maintain a balance (50/50%) between them, depending on the prevailing traffic conditions. In this example, only $\alpha_{circ}$ is considered, since at intersection C0 the dominant movements are associated with the circular artery rather than the radial. The same rationale applies to intersection C2, where circular flows also prevail over radial ones.

(4) $$T=\left\{\begin{array}{c} T_{base}+T_{base}\cdot \;Q\cdot \alpha_{circ/rad/low},\;\;\;\;\rho_{C0-C1}<40\%\\ \;\;\;\;\;\;\;\;\;\;\;\;\;\;\;\;\;\;\;\;\;\;\;\;\;\;\;\;\;\;\;\;T_{base\;\;\;\;\;\;\;\;\;\;\;\;\;\;},\;\;\;\rho_{C0-C1}\geq 40\% \end{array}\right.$$

However, these are not the only percentages considered. A low percentage $\alpha_{low}$, set to 25%, is also introduced for phases that play a minor role at a given intersection. For instance, at intersections C0 and C2, only the $\alpha_{circ}$ factor is applied to phases associated with the circular artery, since the N–S phases at these intersections are relatively marginal and, therefore, receive only a $\alpha_{low\;}$ increment in green time. Conversely, at intersections C3 and C4, the dominant phases correspond to the N–S movements, thus prioritizing the radial artery. In this case, radial phases are extended with a $\alpha_{rad}$ factor, whereas circular phases are assigned only to the lower increment of $\alpha_{low}$.

For intersection C1, both percentages $\alpha_{rad}$ and $\alpha_{circ}$ are considered, as this is the critical intersection and must manage traffic from both arteries according to the allocation defined by the selected strategy. At this intersection, the low percentage $\alpha_{low}$ is applied only to left-turn phases, both for N–S and W–E movements.

In cases where traffic flows from a 400 m link to a 200 m link, the occupancy threshold ρ must be lower. Since the 400 m link can store a large number of vehicles, if the 200 m link has an occupancy below 35%, the corresponding phase is granted its maximum extension. Otherwise, the phase is assigned only to the base duration *T_base_* of 8 s, allowing the 200 m link to reduce its occupancy before receiving additional vehicles.

These thresholds (40%, 65%/35%, 50%/50%, 35%) were empirically calibrated through preliminary simulations to balance traffic flow between radial and circular directions under varying demand. They serve as reference values to trigger phase adjustments, provide stable performance across low- and high-density scenarios, and act as initial heuristic parameters within the SAPA module.

By combining phase selection through neural networks with adaptive phase duration via SAPA module, the system enables more efficient management of both vehicle and pedestrian flows across multiple intersections, ensuring scalability and robustness under diverse traffic conditions. The integration of SAPA with the neural network training further enhances performance by improving traffic fluidity. Specifically, as the average total vehicle waiting time constitutes a component of the reward function, the activation of a heavily demanded phase (e.g., N/S or W/E) no longer corresponds to a fixed duration that may only allow a limited number of vehicles to cross, which would otherwise increase waiting times. Instead, the phase duration is dynamically extended, facilitating greater vehicle mobility and reducing overall delays. This approach not only benefits urban traffic and environmental conditions but also positively influences the agent’s learning process, improving its perception of which strategies maximize vehicular flow.

### 4.4. Traffic Control Strategies Leveraging Arterial Priorities

In order to effectively manage urban traffic, control strategies must account for multiple factors. One of the most relevant aspects concerns rush hours, typically in the morning and in the late afternoon. During the morning peak, the predominant traffic flow is directed into the city, whereas in the afternoon peak the flow is mainly outward. An intelligent traffic control system should, therefore, be capable of identifying the temporal context in which traffic conditions occur by recognizing variations in vehicle flow across specific directions or road segments. With this awareness, the system can prioritize the management of high-volume traffic streams, aiming to maintain overall circulation fluidity, reduce delays and waiting times, and ensure safe mobility across the urban network.

Another critical point is that urban traffic management should not rely on a single control approach, but rather on the integration of at least two complementary mechanisms. The first relates to the exchange of information between agents controlling adjacent intersections. Such communication would allow agents to share data on local traffic states, specifically regarding queue lengths and increases in traffic demand. This information exchange enables each agent to adapt its decision-making process when selecting signal phases. For instance, in the presence of a significant increase in traffic flow along a given road segment, agents can coordinate by adjusting their control strategies. From that moment, and until congestion levels decrease or become stabilized, the system should prioritize the activation of signal phases serving that critical artery, thereby addressing the immediate traffic needs more effectively.

The second mechanism concerns micro-control at the intersection level, particularly in relation to the duration of active signal phases. Since the phases themselves are designed to be adaptive to real-time traffic conditions, it follows that their timing should also be dynamically adjusted. When the adjacent intersection has the capacity to accommodate incoming vehicles, the green phase should be extended in real time, based on the traffic density along the corresponding approach. This functionality, aligned with the role of the previously mentioned SAPA module, would increase the throughput of vehicles at intersections while reducing waiting times. However, such adjustments must be implemented carefully to avoid placing excessive pressure on the downstream intersection, thereby ensuring balanced and coordinated traffic management across the network.

Building on this framework, five distinct traffic control strategies were designed and implemented, with each strategy modeled by a dedicated neural network. These strategies, which are summarized in Table 1, provide complementary approaches to addressing the variability and complexity of urban traffic dynamics. These strategies differ in how they bias the allocation of green phases across intersections, reflecting different priorities between the circular (horizontal) and radial (vertical) arteries, as well as between inbound and outbound flows relative to the central intersection (C1). The objective is to compare a balanced approach with schemes that emphasize one road or traffic direction.

Each control strategy is defined by a specific assumption on urban traffic demand. Strategies may prioritize either the circular road or the radial arteries and further distinguish between outbound (from the center) and inbound (toward the center) flows. These priorities were implemented in the simulation by adjusting the vehicle generation process. For example, and considering a total traffic demand of 1800 vehicles, in all strategies under consideration, 75% of the vehicles (1350 vehicles) proceed straight ahead or make right turns, while the remaining 25% (450 vehicles) correspond to left-turn movements.

In Strategies 2 and 3 of the 1350 vehicles that proceed straight or turn right, 65% (878 vehicles) are generated on the circular artery, whereas the remaining 35% (472 vehicles) originate from the radial artery. To represent inbound and outbound city movements, traffic generation on the radial artery is deliberately unbalanced: 75% of these 472 vehicles (354 vehicles) are produced in the south–north direction (outbound flow) at intersection C4, with the remaining 25% (118 vehicles) generated at intersection C3, or conversely in the case of inbound flows.

Similarly, in Strategies 4 and 5, the same distribution logic applies, but with 65% of the 1350 vehicles (878 vehicles) generated on the radial artery. Again, this generation is unbalanced to simulate inbound and outbound flows, such that 75% (659 vehicles) are generated at C4 and 25% (219 vehicles) at C3. The circular artery, in turn, accommodates the remaining 35% of the 1350 vehicles (472 vehicles).

The experimental setup considered traffic flows of 1800 vehicles and 2000 pedestrians per hour, simulated across 200 episodes of 3600 s each. The vehicle mobility model developed for each strategy captures realistic urban traffic dynamics by considering vehicle speed, acceleration and deceleration patterns, lane-changing behavior, and intersection arrival rates. Additionally, environmental factors such as pedestrian flows, signal timings, road geometry, and variable traffic demand are incorporated to reflect real-world operating conditions. This comprehensive formulation enables the assessment of the DRL agents and the SAPA module under dynamic and realistic traffic scenarios.

The performance of the proposed strategies is evaluated by examining their influence on MARL training and testing processes. Specifically, we compare the final cumulative rewards achieved by each neural network, alongside key traffic efficiency indicators such as average waiting time and throughput.

## 5. Results and Discussion

### 5.1. Performance Evaluation of MARL Training

For the implementation of the five developed traffic control strategies, ten neural networks were trained with two primary objectives. The first was to evaluate traffic optimization performance, with each network being individually trained for a specific traffic generation scenario associated with a given strategy. The second objective was to assess the impact of incorporating the SAPA block, designed to extend the duration of traffic signal phases. In this context, five networks were trained with the inclusion of the SAPA block, while the remaining networks operated under a fixed phase duration of 8 s. However, a fixed duration does not strictly imply that the phase lasts only 8 s. If, after this period, the agent selects the same phase again, its duration is extended for an additional 8 s. Thus, even in the fixed-time configuration, phase extensions may occur through consecutive action selections, although without any explicit adaptation to real-time traffic conditions. Comparisons between these models are, therefore, conducted to analyze the impact of phase duration, both during the training process and in their testing outcomes. All training and testing procedures were performed under a low-traffic scenario, consisting of 1800 vehicles and 2000 pedestrians.

In essence, the study contrasts two distinct concepts. The first entails the activation of a phase with a fixed and relatively short duration—specifically 8 s—thereby increasing the frequency of green signal activations. While this may present certain advantages, its limitation lies in the restricted number of vehicles that can pass during such a short interval. Although the waiting queue is gradually reduced, downstream lanes often have the capacity to accommodate more vehicles than those discharged within the 8 s window. As a result, the potential traffic throughput of the intersection is not fully exploited. With a fixed 8 s phase, the system applies more frequent and localized control within the traffic cell, which may alleviate pressure at the critical intersection—namely, C1—that aggregates inflows from neighboring junctions. However, from an environmental standpoint, the frequent alternation of short green phases promotes stop-and-go driving patterns, increasing fuel consumption and pollutant emissions.

The second concept relies on adopting longer phase durations that adapt to the number of vehicles waiting at a given approach. Extending the green phase increases vehicle throughput at intersections, thereby improving average speeds, reducing queue lengths, and lowering waiting times. This extension must, however, be applied in a controlled manner, requiring information not only about the current approach but also about the downstream section to which vehicles are directed. If the downstream lane is heavily congested, the outflow from the intersection must be limited; conversely, if sufficient capacity is available, throughput can be increased by lengthening the green phase.

**Figure 7:**
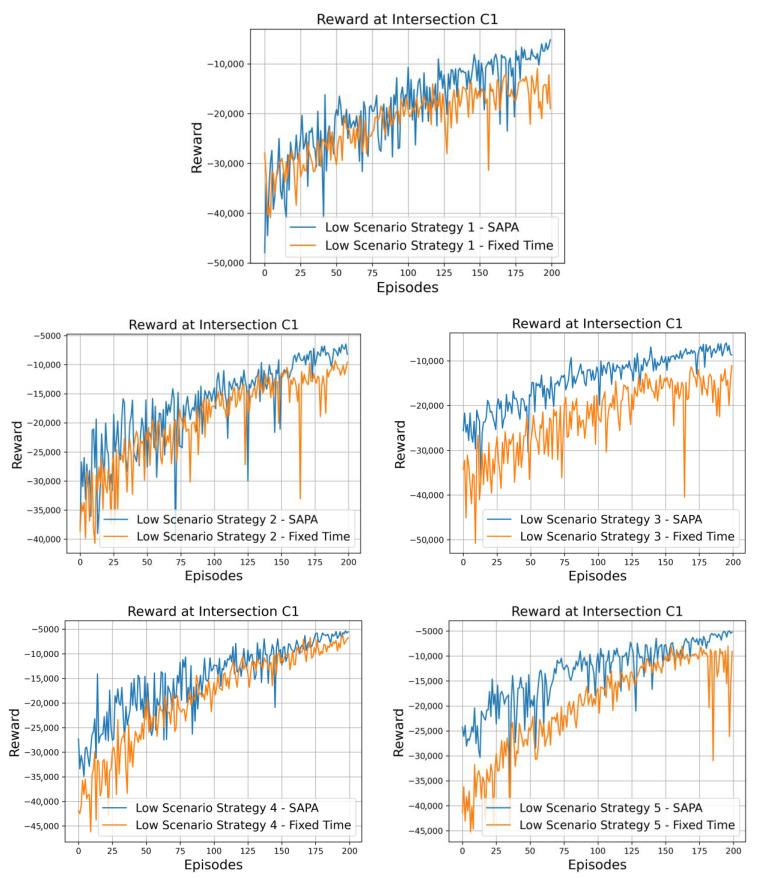
Cumulative negative rewards obtained for both network configurations across each of the strategies, as observed at the critical intersection C1.

Considering these aspects, attention is now directed to Figure 7, which illustrates the rewards obtained for all strategies under both configurations, at the critical intersection C1, with and without the SAPA block. It can be observed that all networks exhibit satisfactory training behavior, displaying a positive slope in reward progression across episodes. Nonetheless, a clear distinction emerges between the two configurations, with the networks incorporating the SAPA block consistently achieving higher rewards. Since the reward is defined in terms of vehicle and pedestrian waiting times, this outcome indicates that networks with the SAPA block encounter fewer difficulties during training, resulting in reduced delays and shorter queues.

Subsequently, the analysis turns to the outputs of the networks for the five strategies under consideration.

### 5.2. Vehicle Queue Dynamics Across the Proposed Strategies

During training, each network learns to interact with the environment, handling a specific traffic generation scenario corresponding to its designated strategy. To verify proper network performance, the models are tested across the different strategies, with the analysis initially focusing on vehicle traffic flow through the intersections of the considered environment. This allows for the observation of the various differences between networks and strategies.

**Figure 8:**
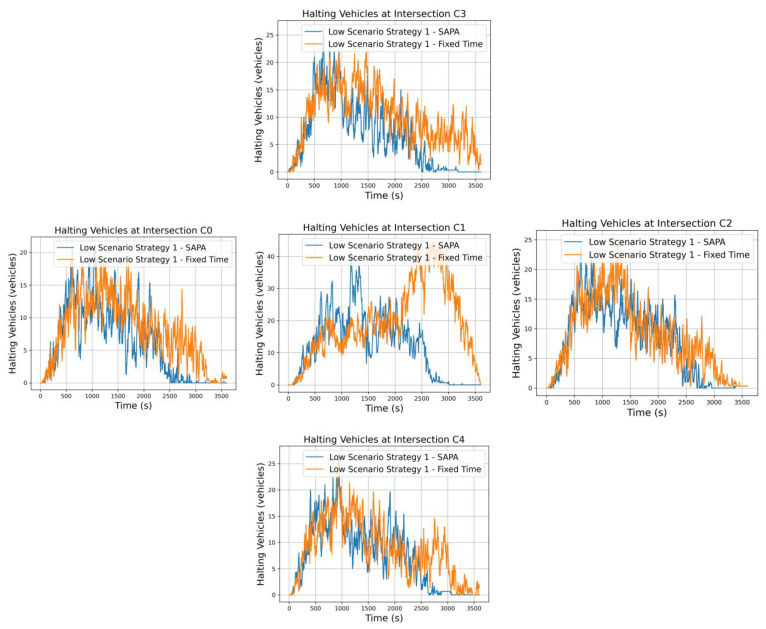
Comparison of vehicle halting at each intersection for both networks under strategy 1.

Starting with strategy 1, illustrated in Figure 8, this approach ensures a balance between radial and circular arteries, resulting in an equilibrium in the activation of N-S and W-E signal phases. Since this strategy does not assign priority to any particular traffic direction, it activates signal phases in a uniformly distributed manner. Consequently, vehicle accumulation increases—most notably at the critical intersection, C1. Nonetheless, this observation does not undermine the utility of the strategy; rather, it fulfills a specific role within the overall configuration of the traffic cell network.

In scenarios where a traffic cell is located at a greater distance from the city center, the adoption of strategy 3, which prioritizes circular traffic flow, may be more appropriate. As vehicles approach the urban core, control measures must become progressively stricter to prevent congestion. Implementing a gradual transition through strategy 1 followed by strategy 5 allows for an incremental prioritization of radial flows, thereby facilitating safer and smoother entry into the city.

A similar rationale applies to outbound traffic. Within the urban core, strategy 4 emphasizes radial flow (e.g., south-to-north). Transitioning to strategy 1 in the subsequent cell introduces a more balanced control, and further along, strategy 2—applied at locations more distant from the center—reestablishes emphasis on circular flow. This strategic sequencing supports the efficient redistribution of traffic, mitigating congestion and enhancing overall flow performance.

It is possible to observe that vehicle halting at intersection C1 aligns with the expected analysis, as traffic at C1 exhibits a higher number of vehicles. Nevertheless, both networks are capable of managing traffic to meet their operational objectives. When comparing the two networks, however, a considerable difference becomes apparent. In the SAPA-enabled network, the average number of vehicles waiting at the peak of the curve is approximately 20, with brief moments reaching 40 vehicles; the network rapidly controls the situation, reducing the queue back to the average. In contrast, the network with fixed times exhibits peaks exceeding 40 vehicles, which continue to increase over time.

These differences are related to phase duration. With SAPA, a single-phase activation can dispatch more vehicles from the intersection, whereas in the network with a fixed 8 s phase duration, multiple activations are required to clear the same number of vehicles. This prolongs the total clearance time and leads to vehicle accumulation at the critical intersection, which is undesirable.

The neighboring intersections C0, C2, C3, and C4 remain relatively balanced, as they are points of vehicle generation and continuously feed traffic into the simulation. Nevertheless, the SAPA network exhibits slightly lower vehicle counts at these intersections due to improved vehicle flow efficiency.

**Figure 9:**
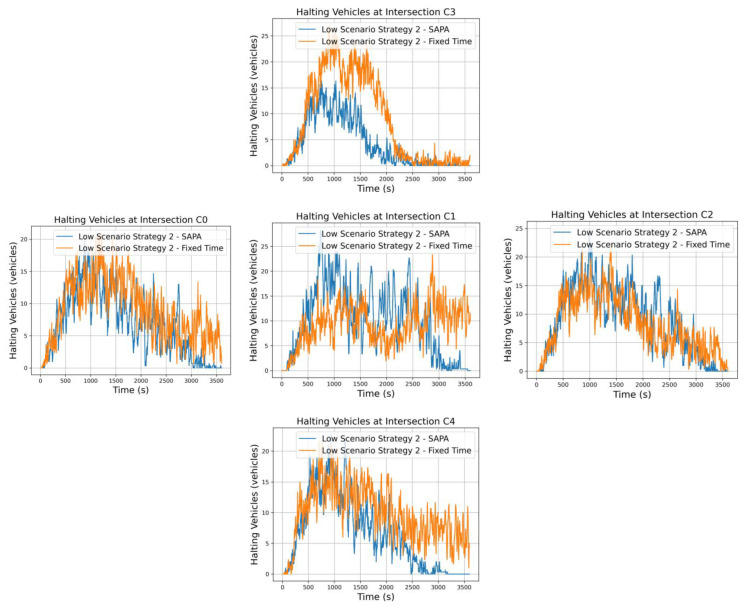
Comparison of vehicle halting at each intersection for both networks under strategy 2.

The analysis then shifts to traffic strategy 2, which prioritizes circular flow at 65% while allocating 35% to radial flow. This configuration targets prominent outbound movements from the city, particularly the S→N traffic between intersections C4 and C3. A higher proportion of vehicles is expected to circulate along the circular artery, prompting the intersection control agents to activate phases associated with this artery more frequently, thereby increasing vehicle flow in these directions. For the radial artery, the strategy emphasizes strong outbound movements from the city, resulting in a higher vehicle accumulation at intersection C4 compared to C3. Figure 9 presents the vehicle halting graphs at the respective intersections for both networks. Focusing on the critical intersection C1, it is possible to observe that the SAPA-enabled network exhibits a higher number of vehicles waiting, which at first glance may seem contradictory. However, this outcome aligns with expectations, as the dynamic phase times allow for greater traffic fluidity across all intersections. This increased flow results in more vehicles passing through intersection C1, naturally leading to longer queues compared to the network with fixed phase durations. Nevertheless, despite the higher vehicle count, C1 remains highly fluid, with sufficient capacity to accommodate additional vehicles before reaching saturation.

Examining the overall vehicle flow, intersections located in storage zones (C0, C2, C3, and C4) generally show a lower number of vehicles waiting. This indicates that dynamic phase durations provided the necessary fluidity and space for vehicles to circulate efficiently.

The particular characteristics of the strategy—circular priority with radial outbound movements—can also be inferred from the graphs. At intersections C1 and C2, the graphs show significant oscillations until a later stage of the simulation, reflecting the continuous movement along the circular artery, with vehicles constantly entering and exiting and gradually decreasing over time. A similar pattern applies to the radial artery. With strong outbound movements, C4 shows a higher number of S→N vehicles entering, while C3 exhibits fewer vehicles. The differences between the networks are significant, highlighting the improved traffic fluidity achieved with SAPA.

Regarding strategy 3, it is similar to strategy 2 in that a higher traffic volume is assigned to the circular artery. However, for the radial artery, the strongest movements are now inbound to the city, specifically N→S from intersection C3 to C4. As in strategy 2, a high vehicle flow is expected along the circular artery, but for the radial artery, inbound movements dominate, resulting in a greater number of vehicles at C3 compared to C4.

**Figure 10:**
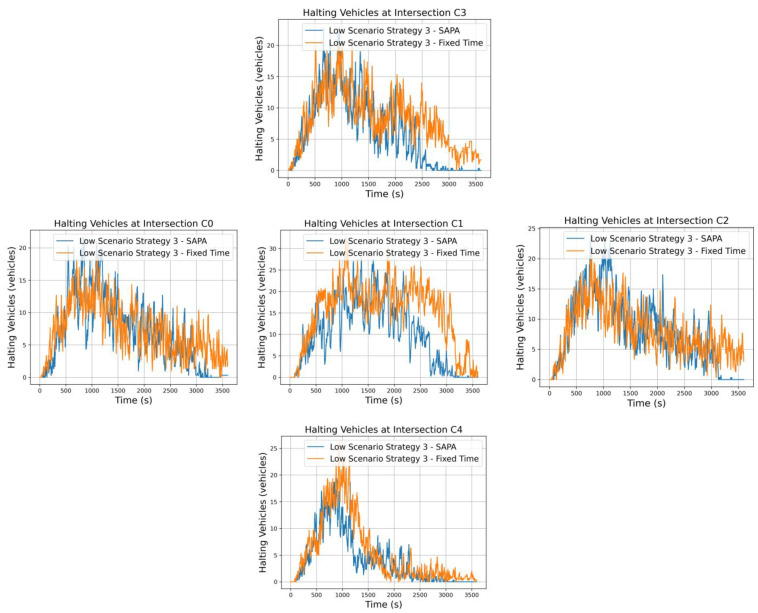
Comparison of vehicle halting at each intersection for both networks under strategy 3.

Analyzing vehicle halting as shown in Figure 10, some improvements are evident for the SAPA network, consistent with the trends previously observed in the reward metrics. Along the circular artery, intersections C0 and C2 exhibit similar behavior across both networks, as a high vehicle flow in this artery results in continuous oscillations, with vehicles entering and exiting the intersections over time.

For the radial artery, the characteristics of the strategy are again apparent: C4 shows fewer vehicles, while C3, being a stronger entry point, accumulates more vehicles over time, gradually decreasing as the simulation progresses. At the critical intersection C1, the SAPA network demonstrates better vehicle control and flow, as expected, leading to an increased average speed within this critical traffic cell area.

Comparing vehicle halting at intersections C3 and C1 for strategies 2 and 3 highlights the differences between the strategies, which are related to the capacities of the respective roads. Both strategies assign a higher volume of vehicles to the circular artery, differing only in the dominant radial movements: strategy 2 emphasizes S→N outbound movements, while strategy 3 emphasizes N→S inbound movements. Comparing C1 halting between the two strategies reveals that this intersection experiences a higher average number of vehicles waiting during inbound movements.

This discrepancy is linked to the road capacities connecting the intersections: the C3–C1 segment is 200 m long, whereas the C4–C1 segment is twice as long at 400 m. The longer C4–C1 road allows vehicles to remain in motion longer before reaching the queue point. In a low-demand scenario with less prioritized radial movements, this route rarely accumulates enough vehicles to exert significant pressure on C1. With SAPA and its extended phase duration, vehicles flow efficiently toward C3. In contrast, for strategy 3 with inbound movements, vehicles travel N→S along the shorter 200 m C3–C1 segment. Due to the shorter length, vehicles reach the queue point sooner, remaining less time in motion, which results in a higher average number of vehicles waiting at C1.

The analysis now shifts to strategies that emphasize a strong radial artery, with a higher vehicle flow compared to the circular artery. For strategy 4, in addition to this arterial imbalance, the strategy also prioritizes stronger outbound movements from the city, specifically, more vehicles traveling from C4 to C3. It is, therefore, expected that circular flow priority at C1 will decrease due to the reduced vehicle flow in these directions, while the radial artery becomes the main focus, emphasizing outbound movements. As a result, a higher number of vehicles is anticipated at C4 compared to C3.

**Figure 11:**
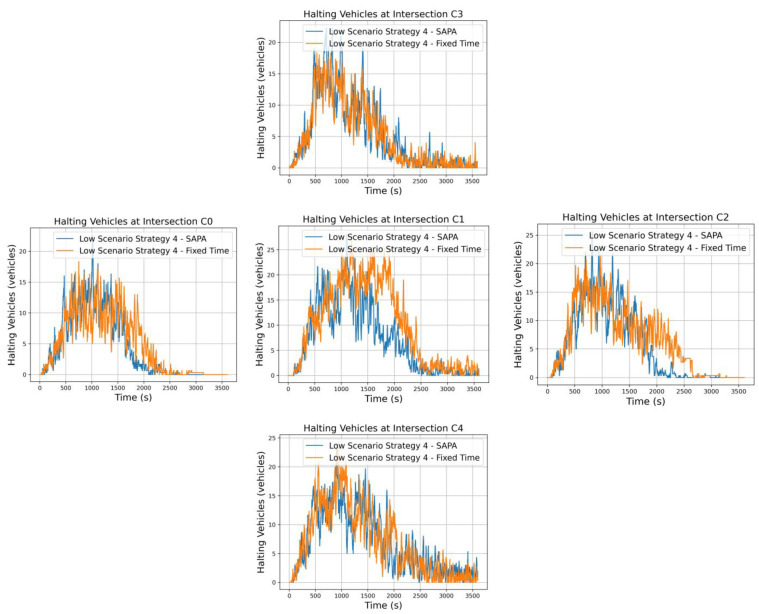
Comparison of vehicle halting at each intersection for both networks under strategy 4.

Figure 11 illustrates vehicle halting at the respective intersections for both networks. The results are generally similar; however, the SAPA-enabled network exhibits slightly fewer vehicles waiting. This difference is most notable at the critical intersection C1, where the peak number of vehicles waiting reaches only 20, compared to slightly over 25 in the network with fixed times. Nevertheless, this critical area remains well-controlled and fluid, which is beneficial for overall traffic conditions and the surrounding environment

Finally, strategy 5 again prioritizes a stronger radial artery over the circular artery, but now with dominant inbound movements to the city, specifically N→S traffic from C3 to C4. Similar behavior is expected along the circular artery; however, for the radial artery, more vehicles are anticipated at C3 than at C4, reflecting the emphasis on inbound city movements.

**Figure 12:**
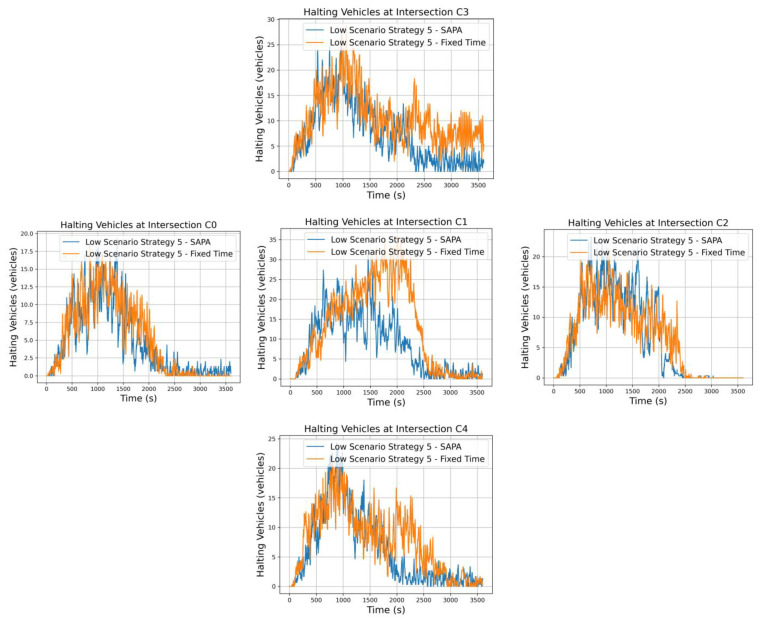
Comparison of vehicle halting at each intersection for both networks under strategy 5.

Figure 12 presents the vehicle halting graphs for each intersection in both networks. The most notable differences between the networks occur along the radial artery, where the highest vehicle volumes circulate and where the SAPA network achieves the best results, with fewer vehicles waiting in queues. As C3 serves as the primary entry point for vehicles into the environment, its queue length fluctuates considerably in both networks. However, with the SAPA block, phase durations are extended when capacity allows on the C3–C1 segment, effectively increasing the flow of vehicles through C3. As a result, vehicle oscillations are roughly half of those observed in the network with fixed times.

At C1, significant improvements are also evident. During peak periods, the average number of vehicles waiting is around 20, indicating a well-controlled intersection with smooth vehicle flow, whereas the network without SAPA shows an average of 30 vehicles in the queue. Similarly, C4 benefits from optimized control, reducing the number of vehicles waiting and improving overall intersection performance.

After analyzing all five strategies, it can be generally concluded that the network incorporating the SAPA block achieved the best overall performance, as expected. By extending the phase duration, traffic flow at the intersections increased, enhancing mobility and reducing queue lengths. Furthermore, for all strategies, the critical intersection C1 exhibited consistently positive results, maintaining smooth traffic flow and avoiding excessive vehicle accumulation, which could otherwise compromise the overall traffic environment.

### 5.3. Evaluation of Pedestrian Flows and Agent-Managed Signal Phases

The analysis of pedestrian traffic is essential to understanding the overall performance of urban intersections, as pedestrian movements interact closely with vehicular flow and signal control strategies. By examining the patterns of pedestrian crossings and the corresponding active phases selected by the agents, it becomes possible to assess how each strategy balances the needs of both vehicles and pedestrians. This assessment provides insights into the efficiency, safety, and adaptability of the implemented traffic control approaches.

**Figure 13:**
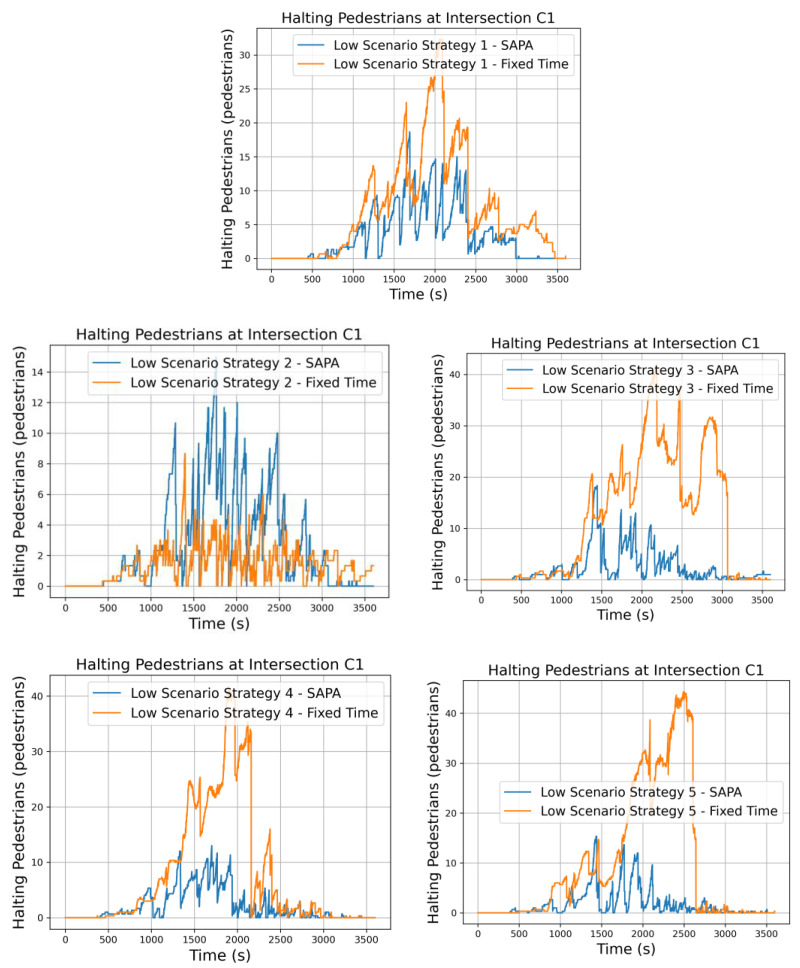
Pedestrian halting in waiting areas at intersection C1 for the five strategies across both networks.

The examination of pedestrian stopping behavior, illustrated in Figure 13, shows that the magnitude of halting peaks serves as a useful indicator of the level of stress experienced at intersections and reflects pedestrians’ interactions with connected vehicles. In general, the stopping levels remain relatively consistent across the five networks, indicating that all strategies are effective in accommodating pedestrian flows without introducing substantial delays. Some temporal variations are noted, mainly linked to the timing of pedestrian phase activations, but these fluctuations do not noticeably affect overall performance. These results imply that pedestrian flows are managed efficiently, causing minimal disruption to both pedestrian and vehicular traffic, thereby highlighting the robustness of the proposed network control strategies. As vehicles also yield to pedestrians, the number of pedestrians waiting in designated areas decreases for the network incorporating the SAPA block. With a smoother traffic environment and reduced vehicle congestion, the agent at each intersection has more opportunities to activate pedestrian phases, thereby lowering the number of waiting pedestrians.

**Figure 14:**
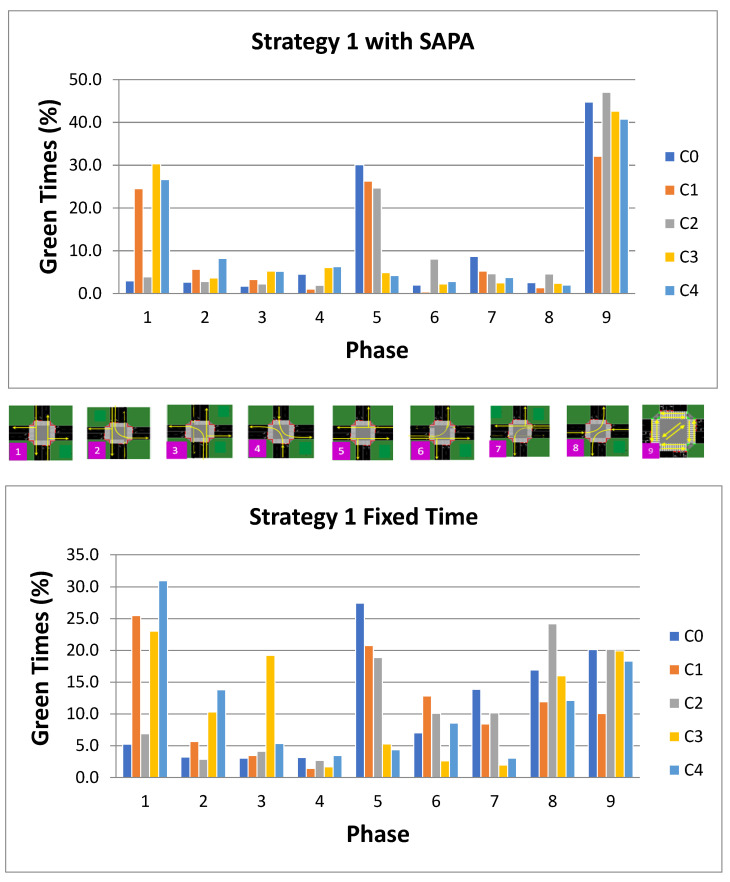
Percentage of active phases over the simulation time for each intersection (C0–C4) under Strategy 1, comparing the configurations with and without the SAPA module.

The analysis now turns to the active phases executed by the agents at each intersection in both neural networks. For Strategy 1, both arterial directions should remain balanced, meaning that the active phases in the N–S direction should correspond closely to those in the W–E direction. Figure 14 illustrates the active phases executed by the agents at each intersection over time for both neural networks under study.

**Figure 15:**
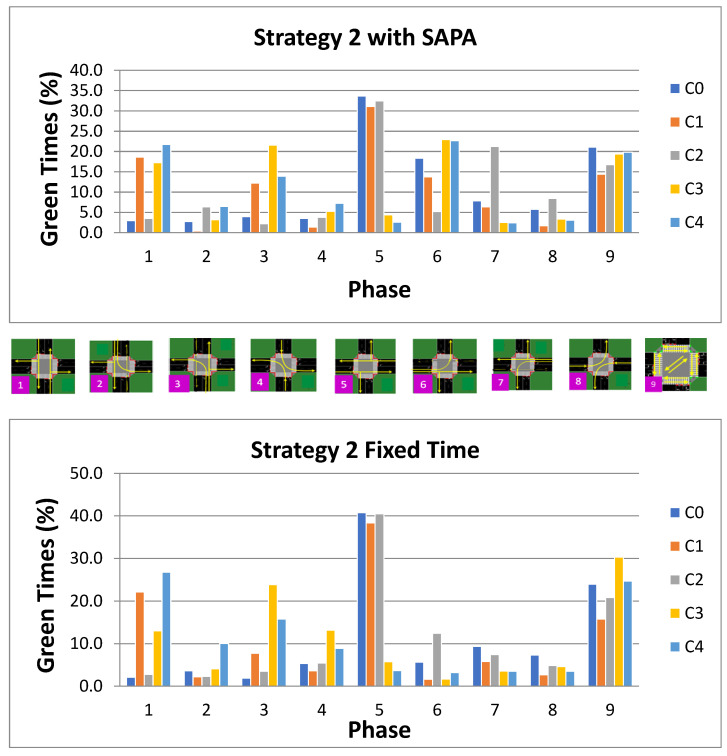
Percentage of active phases over the simulation time for each intersection (C0–C4) under Strategy 2, comparing the configurations with and without the SAPA module.

Overall, it can be observed that the dominant phases are P1 (N–S), P5 (W–E), and P9 (pedestrian phase). A notable difference between the two networks is that the weaker phases in the N–S direction (P2, P3, and P4) and W–E direction (P6, P7, and P8) are less frequently activated in the SAPA network. These weaker phases are largely replaced by pedestrian phase activations, which show an increase of over 20% in the SAPA network compared to the baseline. Nonetheless, both networks maintain an approximately balanced activation between P1 and P5 at intersection C1. For Strategy 2, the activation patterns of phases at the intersections, as managed by the agents, are illustrated in Figure 15. A higher activation of phase P5 (W–E) is expected to accommodate the circular artery, where vehicle volumes are higher, thereby maintaining a steady traffic flow. In contrast, the radial artery experiences increased flow from S→N (i.e., from C4 to C3), simulating outbound traffic from the city.

Analysis of the figure indicates that both networks exhibit similar percentages of phase activation. Phase P1 is more prominent at intersections C1, C3, and C4, ranging between 13% and 26%, which represents a decrease compared to Strategy 1, where it varied between 23% and 30%. Another notable N–S phase is P3 (S–ALL), which shows a significant increase, ranging from 7% to 24%. With fewer vehicles on the radial artery under the low-traffic scenario (1800 vehicles), N→S vehicles, particularly those generated at C3, are depleted more rapidly, as reflected in Figure 9, where C3 consistently exhibits the lowest number of waiting vehicles. This allows the network to prioritize phase P3, enabling the full movement of vehicles from S→N, explaining the observed increase in its activation percentage for this strategy.

Phase P5 varies between 30% and 40%, indicating efficient traffic flow along the circular artery due to its high vehicle volume. This also leads to increased activations of phases P6 and P7 (W–ALL and E–ALL), reflecting the network’s adaptive response to manage traffic in these directions.

**Figure 16:**
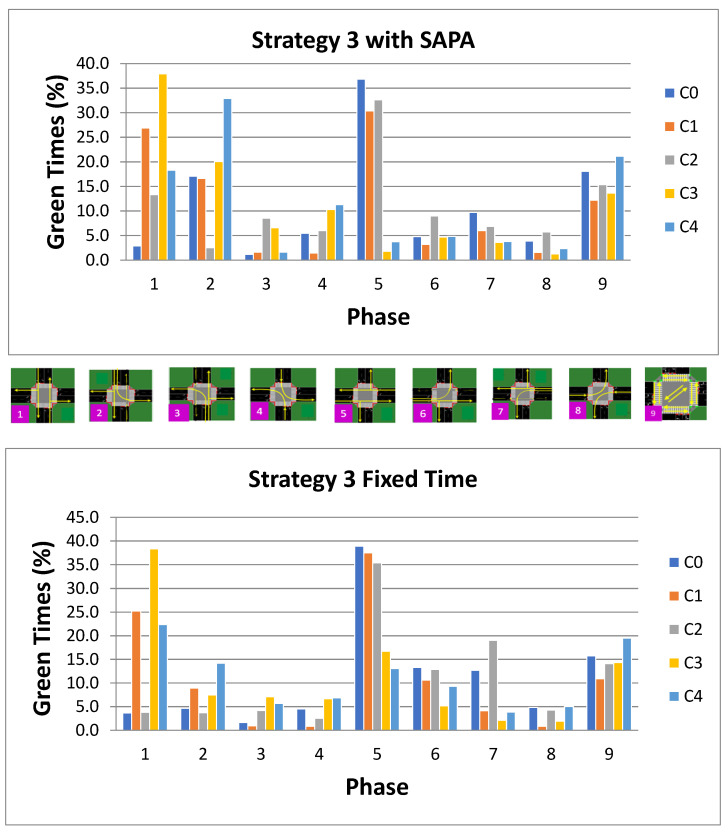
Percentage of active phases over the simulation time for each intersection (C0–C4) under Strategy 3, comparing the configurations with and without the SAPA module.

For Strategy 3, which is similar to Strategy 2 but emphasizes strong inbound movements into the city, the activation patterns of phases at each intersection are illustrated in Figure 16. A substantial activation of phase P5 (W–E) is observed at intersections along the circular artery, as expected, resulting in increased vehicle flow along these routes.

For the radial artery, particularly in the network incorporating the SAPA block, high percentages of phase P1 (N–S) and phase P2 (N–ALL) are observed. The increased activation of phase P1 is directly related to the characteristics of this strategy, which assumes a weak radial artery but strong inbound traffic from N→S. Pedestrian phase activation remains largely similar across both networks.

**Figure 17:**
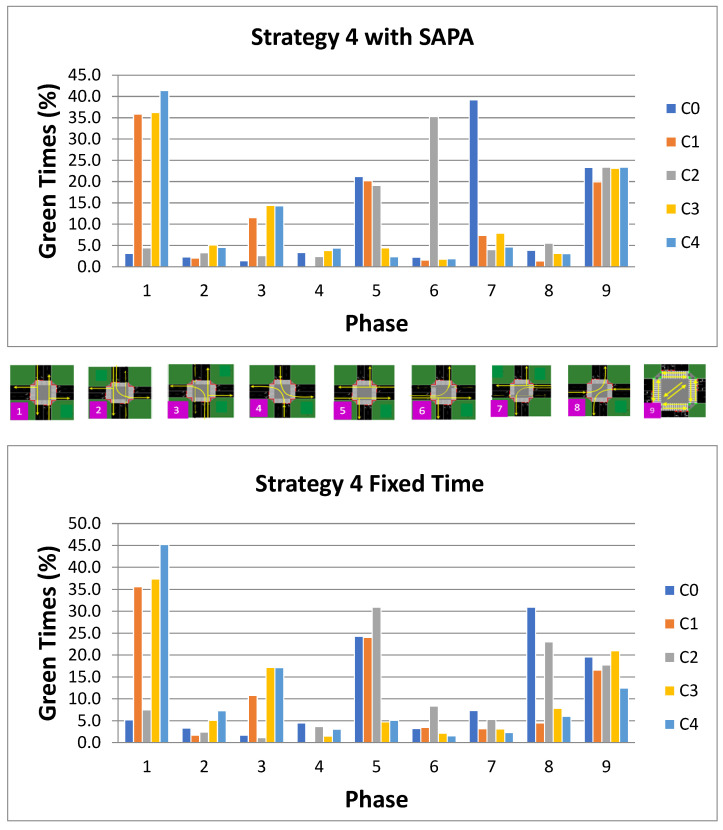
Percentage of active phases over the simulation time for each intersection (C0–C4) under Strategy 4, comparing the configurations with and without the SAPA module.

For strategy 4, Figure 17 depicts the temporal distribution of phase activations at each intersection across both networks, with and without the SAPA block. The observed activation patterns reflect the inherent characteristics of this strategy, as phase P1 (N–S) exhibits higher activation percentages than phase P5 (W–E), indicating a greater vehicle flow along the radial artery compared to the circular artery. Phase P3 (S–ALL) also demonstrates an increased activation, ranging from 11% to 14%.

A comparison between the two outbound strategies, Strategies 2 and 4, reveals that phase P3 is less frequently activated under conditions of higher traffic volumes along the radial artery. Overall, the radial artery experiences elevated bidirectional flow relative to Strategy 2, which enhances the prioritization of phase P1 (N–S) and consequently increases vehicle throughput at the corresponding intersections. Nevertheless, phase P3 remains among the most frequently utilized phases in scenarios characterized by strong outbound movements. Furthermore, pedestrian phase activation is consistently higher in the SAPA network, contributing to a reduction in the number of pedestrians waiting at designated areas.

**Figure 18:**
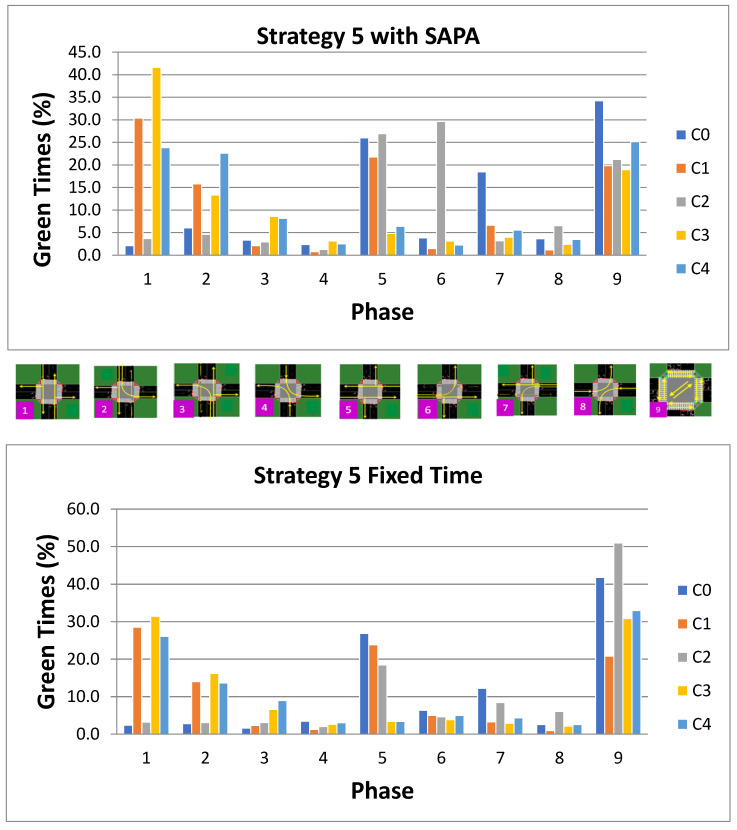
Percentage of active phases over the simulation time for each intersection (C0–C4) under Strategy 5, comparing the configurations with and without the SAPA module.

Finally, for Strategy 5, Figure 18 presents the percentages of active phases executed by the agents at each intersection for both networks. The phase activation patterns reflect the characteristics of this strategy, as phase P1 (N–S) exhibits a higher activation percentage compared to phase P5 (W–E), consistent with the increased vehicle flow along the radial artery.

An increase in the activation of phase P2 (N–ALL), ranging between 13% and 22%, is also observed, as this strategy emphasizes strong inbound traffic into the city. When compared to Strategy 3, which also focuses on city entry but with lower vehicle volumes on the radial artery, phase P2 shows higher activation in the SAPA network, varying between 17% and 32%. For Strategy 5, however, the activation percentage of this phase decreases. This adjustment is directly related to the increased number of vehicles on the radial artery, making it more advantageous to prioritize phase P1 (N–S) to facilitate bidirectional traffic flow along this artery.

### 5.4. Comparative Analysis of Traffic Flow Performance

**Figure 19:**
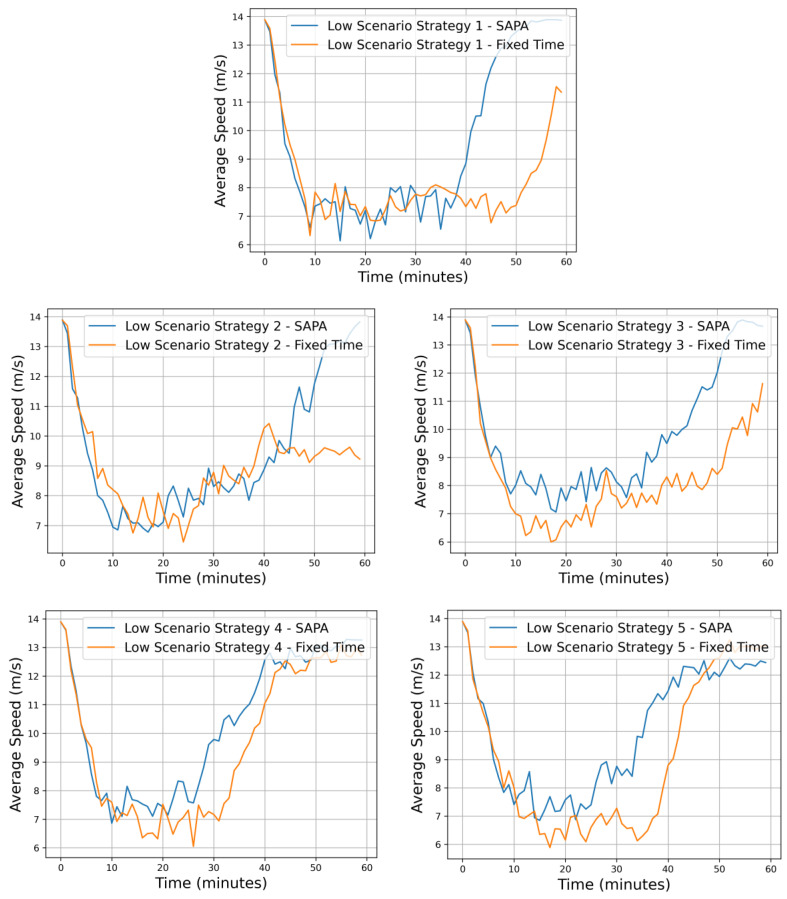
Average speeds at critical intersections (C1) of the urban environment for each traffic control strategy under study.

So far, improvements have been observed both in terms of the reward function and the halting times of vehicles and pedestrians with the inclusion of the SAPA module. The adaptive adjustment of signal phase durations to current traffic conditions—considering both vehicle flows and the occupancy of queues at adjacent intersections—has led to a reduction in the number of vehicles waiting in queues. By extending the phase duration, vehicles benefit from a larger time window in which, if conditions allow, they can remain in motion, thereby enhancing overall traffic fluidity. To quantitatively illustrate this improvement, particularly in the critical area of the environment corresponding to intersection C1, Figure 19 presents the average vehicle speed over the one-hour simulation period. The results indicate that the SAPA-enhanced network not only reduces congestion at bottlenecks but also improves the efficiency and responsiveness of traffic flow, demonstrating the potential of adaptive phase management in urban traffic control systems.

For Strategy 1, it is possible to observe that during the peak period between 10 and 40 min, average speeds across the arterial roads are similar, reflecting the complexity of simultaneously controlling both routes. After the peak period, speeds increase considerably for the SAPA-enhanced network. A similar pattern is observed for Strategy 2, where notable differences persist only until approximately 45 min. In contrast, for the remaining strategies, the differences between the baseline and SAPA networks are pronounced from the early stages of the simulation. These observations clearly indicate that prioritizing one arterial road, in combination with adaptive phase duration adjustments, leads to a significant improvement in traffic fluidity, particularly in critical areas of the network.

## 6. Conclusions

Conventional traffic control systems that rely on cyclic activation of fixed-time phases remain one of the major limitations of current urban traffic management. Their inability to adapt phase activation to real-time traffic conditions results in longer queues, increased delays, and inefficient use of road capacity. This study addresses these limitations by presenting an intelligent and adaptive traffic control framework capable of optimizing active phases at intersections using real-time data.

A central scientific contribution of this work lies in the integration of a decentralized Multi-Agent Reinforcement Learning (MARL) framework with a fully connected V2X environment enabled by Visible Light Communication (VLC). The system leverages VLC-based sensing—an element seldom explored in combination with DRL approaches—to obtain high-resolution, low-latency traffic information from vehicles, pedestrians, and infrastructure. This real-time perception feeds a set of decentralized agents that jointly manage a traffic cell composed of five intersections under diverse urban mobility scenarios, including arterial, circular, and radial flows.

Another key innovation introduced in this study is the Strategic Anti-Blocking Phase Adjustment (SAPA) module, designed to dynamically adapt phase durations according to queue formation and the downstream storage capacity of adjacent intersections. This mechanism directly targets one of the major shortcomings of existing DRL-based traffic control approaches: their limited ability to anticipate blocking propagation between intersections.

Experimental results confirm that the combined MARL + SAPA architecture yields superior performance compared to isolated components, reducing vehicle idling times, improving overall traffic fluidity, and increasing pedestrian safety through the use of an exclusive pedestrian phase. The analysis of agent-selected phases further demonstrates the system’s capacity to adapt to multiple traffic generation patterns, validating its potential as a scalable, decentralized, and intelligent solution for complex urban environments. These findings substantiate the scientific novelty of integrating VLC-enabled perception with adaptive DRL-based control, contributing a new methodological perspective for multi-intersection traffic management.

Despite these results, several limitations must be acknowledged. The simulations assume ideal VLC without failures or environmental disturbances, and full penetration of VLC-enabled devices in all vehicles—conditions not yet fully representative of real urban settings. Overcoming heterogeneous communication capabilities and ensuring robust system performance under imperfect sensing conditions will require additional mechanisms and fault-tolerance strategies.

Future work will include the study of interactions between multiple traffic cells operating under distinct strategies, driven by metrics such as time of day, queue dynamics, and waiting times. Extending the framework toward city-wide coordination will further explore its scalability and support real-world deployment of adaptive, VLC-enhanced traffic control systems.

## Figures and Tables

**Table 1:** Summary of traffic control strategies applied.

Network	Strategy	Priority Artery	Direction Focus	Description
1	Standard	None	W-E N-S	Arteries and directions treated equally.
2	Circular + Outbound Radial	Circular	E-W Northbound (C4 → C3)	Prioritizes circular artery with outbound radial flow (S→N).
3	Circular + Inbound Radial	Circular	E-W Southbound (C3 → C4)	Prioritizes circular artery with inbound radial flow (N→S).
4	Radial + Outbound Radial	Radial	N-S Northbound (C4 → C3)	Prioritizes radial artery with outbound radial flow.
5	Radial + Inbound Radial	Radial	N-S Southbound (C4 → C3)	Prioritizes radial artery with inbound radial flow.

## Data Availability

The original contributions presented in this study are included in the article. Further inquiries can be directed to the corresponding author(s).
